# Transcriptome Profiling of *Louisiana iris* Root and Identification of Genes Involved in Lead-Stress Response

**DOI:** 10.3390/ijms161226084

**Published:** 2015-11-25

**Authors:** Songqing Tian, Chunsun Gu, Liangqin Liu, Xudong Zhu, Yanhai Zhao, Suzhen Huang

**Affiliations:** 1 Institute of Botany, Jiangsu Province and Chinese Academy of Science, Nanjing 210014, China; 2012204030@njau.edu.cn (S.T.); chunsungu@cnbg.net (C.G.); 2010204026@njau.edu.cn (X.Z.); 2014216028@njau.edu.cn (Y.Z.); 2 Suzhou Polytechnical Institute of Agriculture, Suzhou 215008, China; 3 College of Horticulture, Nanjing Agricultural University, Nanjing 210014, China; 2013104113@njau.edu.cn

**Keywords:** *Iris*, Lead, transcriptome, RT-qPCR

## Abstract

*Louisiana iris* is tolerant to and accumulates the heavy metal lead (Pb). However, there is limited knowledge of the molecular mechanisms behind this feature. We describe the transcriptome of *Louisiana iris* using Illumina sequencing technology. The root transcriptome of *Louisiana iris* under control and Pb-stress conditions was sequenced. Overall, 525,498 transcripts representing 313,958 unigenes were assembled using the clean raw reads. Among them, 43,015 unigenes were annotated and their functions classified using the euKaryotic Orthologous Groups (KOG) database. They were divided into 25 molecular families. In the Gene Ontology (GO) database, 50,174 unigenes were categorized into three GO trees (molecular function, cellular component and biological process). After analysis of differentially expressed genes, some Pb-stress-related genes were selected, including biosynthesis genes of chelating compounds, metal transporters, transcription factors and antioxidant-related genes. This study not only lays a foundation for further studies on differential genes under Pb stress, but also facilitates the molecular breeding of *Louisiana iris*.

## 1. Introduction

The contamination of water by toxic heavy metals can occur naturally during soil erosion and flooding or anthropogenically by automobile emissions of leaded gasoline, emissions from industrial factories of coal combustion, smelting and cement production, agrochemicals, insecticides and herbicides [1,2]. Lead (Pb) is a toxic heavy metal. The contamination of lakes, rivers, domestic waters and other water sources by Pb may lead to ecotoxicological problems in plants and animals, as well as damage to human health [3]. Plants could be effective heavy metal biological monitors, remediating contamination through rhizofiltration, phytostabilization or phytoextraction [4]. Therefore, identifying Pb hyperaccumulator plants, and the study of Pb absorption, transport and tolerance mechanisms in such plants, is very important.

*Louisiana iris* is an ornamental water plant, having originated in the marshes and swamps of the Gulf Coast States in the USA. Their natural habitat is comprised of wet, boggy areas that flood during the winter and spring [5]. New *Louisiana iris* cultivars grow well in various environmental uplands and wetlands, and are the most colorful of all of the *Iris* groups, making it a popular landscape plant. *I. hexagona* is one of its parents, and the *I. hexagona* population has become exposed to salt stress for more than 2000 years in southern Louisiana [6].

In recent years, RNA deep-sequencing (RNA-seq) has been used on several plants [7], including soybean [8], maize [9], cotton [10], chickpea [11], Chinese cabbage [12], potato [13], and *Ammopiptanthus mongolicus* [14]. It is a powerful method to screen for genes under biotic and abiotic stress conditions. Moreover, gene regulatory networks can be determined by RNA-seq. Some RNA-seq analyses of responses to Pb stress in plants have been reported. In the radish root, many differentially expressed genes (DEGs), which participated in defense and the detoxification of Pb, were identified [15]. In response to Pb treatments, the transcriptional profiles of over 1310 genes in common stress responses or different biological pathways were investigated in *Arabidopsis thaliana* [16]. These studies indicated that plants have formed a complete response and defense system through a series of regulatory genes in response to Pb stress.

In previous studies, *I. tectorum*, *I. lactea* var. *chinesis* and *I. halophila* were found to be resistant to heavy metals [17,18,19]. *Louisiana iris* was found to be tolerant to and accumulate Pb in our physiological studies [20]. In *Iris* research, the transcriptomes of floral and young leaf tissue from *I. fulva* have been described [5]. However, there are no reports of genetic or genomic information for *Louisiana iris*. In our study, we used Illumina sequencing technology to facilitate the understanding of Pb tolerance in *Louisiana iris* at a molecular regulatory level.

## 2. Results and Discussion

### 2.1. Sequencing and Reads Assembly

**Figure 1:**
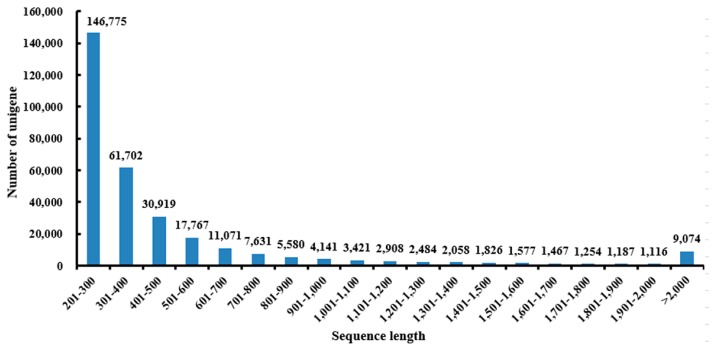
The length distribution of the assembled unigenes.

When plants are under heavy metal stress, there may be an intense metabolism modulation by 24 h [15,20]. Thus, a 24-h time course of Pb stress was used to characterize the Pb-responsive genes in our study. To obtain a global overview of the *Louisiana iris* gene expression profile under Pb-stress conditions, cDNAs from roots of control and Pb stressed *Louisiana iris* were sequenced using the Illumina HiSeq™ 2000 platform. We obtained approximately 71 million raw reads from the 200 mg/L^−1^ Pb-treated sample and 80 million raw reads from the control sample, and we removed low-complexity, low-quality and repeat reads. After strict quality checks and the cleaning of data, we had more than 128 million valid reads containing more than 12.0 G of nucleotides. The average read size and read valid ratio were 93.84 bp and 85.32%, respectively. After assembly, adaptor tags and repeat reads were filtered out, producing 525,498 transcripts, and the mean transcript size was 726 bp. After clustering using chrysalis clusters, the lengths of the max contig, min contig, whole dataset, average contig and N50 were 15,986, 205, 156,000,000, 497 and 572 bp, respectively, in 313,958 unigenes (Table 1). The length distribution of the assembled unigenes indicated that 239,396 (76.25%) unigenes ranged from 201 to 500 bp in length; 46,190 (14.71%) unigenes ranged from 501 to 1000 bp in length; 19,298 (6.15%) unigenes ranged from 1001 to 2000 bp in length; and 9074 (2.89%) unigenes were more than 2000 bp in length (Figure 1). During assembly, 7.42% of the unigene annotations matching genes in *Phytophthora sojae*, *Tetrahymena thermophila*, *Albugo laibachii* Nc14, *Paramecium tetraurelia* and *Phytophthorain festans* were found. Interestingly, many more unigenes matched to *Phytophthora sojae* than to others in “non-plant” organisms. The diseases caused by *Phytophthora* in *Iris* occurred from the early growing season until the flowering stage [21]. The *Iris* plant used in this experiment may have been infected by *Phytophthora*. The disease cannot be phenotypically detected by the naked eye. This may be why a significant proportion of the unigenes were annotated as *Phytophthora sojae*. Fortunately, this result could also help explain Pb-stress biology and is therefore still meaningful to our study.

**Table 1:** Overview of the sequencing and assembly.

Items	Number
Total unigenes	313,958
Max contig length (bp)	15,986
Min contig length (bp)	205
Whole dataset length (bp)	156,000,000
Average contig length (bp)	497
N50	572

### 2.2. Annotation of Unigenes

**Figure 2:**
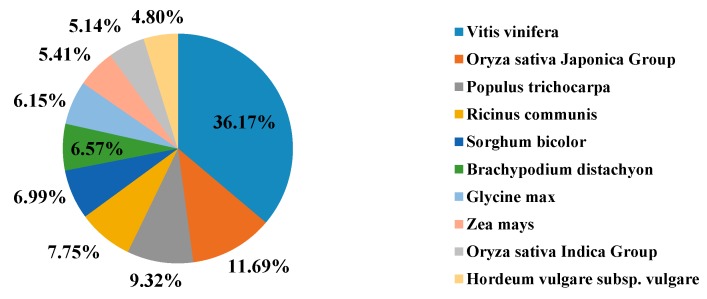
The most represented species distribution.

**Figure 3:**
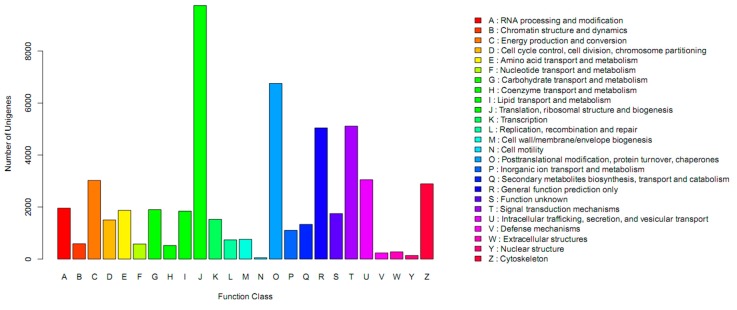
The GO classification of the assembled transcripts.

**Figure 4:**
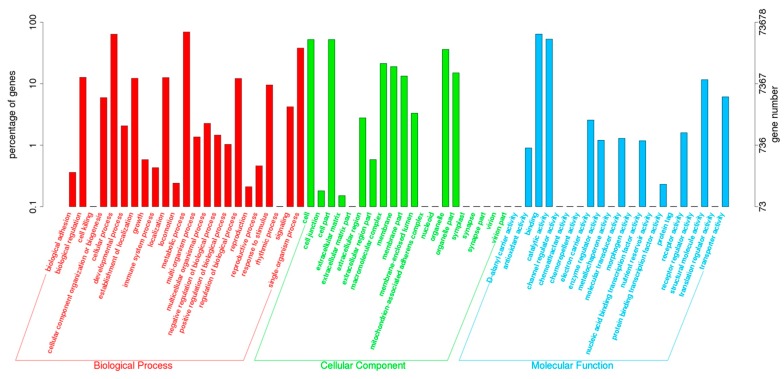
Histogram of KOG classifications.

All of the unigenes were aligned against protein databases of Nr, SWISS-PROT, TrEMBL, Conserved Domain Database (CDD), Protein Families Database of Alignments and Hidden Markov Models (PFAM) and Eukaryotic orthologous groups (KOG). The number of aligned unigenes was 89,738 (28.58%) in Nr, 61,342 (19.54%) in SWISS-PROT, 93,087 (29.65%) in TrEMBL, 55,710 (17.74%) in CDD, 80,756 (25.72%) in PFAM and 43,015 (13.70%) in KOG (Table 2). In the Nr database, the top 10 species annotated were Vitis vinifera (36.17%), Oryza sativa Japonica Group (11.69%), Populus trichocarpa (9.32%), Ricinus communis (7.75%), Sorghum bicolor (6.99%), Brachypodium distachyon (6.57%), Glycine max (6.15%), Zea mays (5.41%), Oryza sativa Indica Group (5.14%) and Hordeum vulgare subsp. Vulgare (4.80%) (Figure 2). In the KOG database, 43,015 unigenes were classified into 25 functional categories (Figure 3). In the Gene ontology (GO) database, 50,174 unigenes were classified into the three ontologies, biological process, cellular component and molecular function (Figure 4). For biological process, “cellular process” and “metabolic process” had the highest numbers of isotigs. For cellular component, “cell” and “cell part” had the highest numbers of isotigs. For molecular function, “binding” and “catalytic activity” had the highest numbers of isotigs (Figure 4).

**Table 2:** List of annotations.

Annotation Database	Number of Annotations	Percent of Annotation (%)
Total unigene	313,958	-
Nr	89,738	28.58%
SWISS-PROT	61,342	19.54%
TrEMBL	93,087	29.65%
CDD	55,710	17.74%
PFAM	80,756	25.72%
KOG	43,015	13.70%

“-” means no percent of annotation.

At the Bonferroni-corrected alpha level of *p* ≤ 0.05, 30,772 up-regulated DEGs were categorized into 804 GO terms (Table S1), and 29,990 DEGs involved in 720 predicted GO terms were significantly down-regulated (Table S2). The Pb-response genes were mapped to the Kyoto Encyclopedia of Genes and Genomes (KEGG) database. Among the enriched pathways of up-regulated genes (Table 3), the KEGG pathway analysis found 13,204 unigenes involved in 52 predicted KEGG metabolic pathways. Additionally, 15,666 unigenes were involved in 102 predicted KEGG metabolic pathways among the pathways enriched with down-regulated genes (Table 4). Partial pathways are shown in Table 3 and Table 4. Compared to the results in radish [15], the metabolism of xenobiotics by cytochrome P450 [ko00980] and gap junctions [ko04540] were also up- and down-regulated pathways, respectively, under Pb stress. The results suggest that the same regulatory pathways function in different species under Pb stress.

**Table 3:** Enriched pathways containing up-regulated genes under Pb stress.

Pathway	Id	Number of DEGs with Pathway Annotations (13,204)	Number of Genes with Pathway Annotations (14,105)	*p*-Value
Metabolism of xenobiotics by cytochrome P450	ko00980	134 (1.02%)	287 (2.03%)	3.06 × 10^−8^
Drug metabolism-cytochrome P450	ko00982	122 (0.92%)	267 (1.89%)	4.53 × 10^−7^
PPAR signaling pathway	ko03320	221 (1.67%)	548 (3.89%)	9.40 × 10^−7^
Protein processing in endoplasmic reticulum	ko04141	796 (6.03%)	2332 (16.53%)	8.32 × 10^−6^
Ribosome biogenesis in eukaryotes	ko03008	264 (2.00%)	705 (5.00%)	5.85 × 10^−5^
RNA polymerase	ko03020	122 (0.92%)	297 (2.11%)	1.94 × 10^−4^
Ubiquitin mediated proteolysis	ko04120	392 (2.97%)	1123 (7.06%)	5.06 × 10^−4^
Proteasome	ko03050	313 (2.37%)	886 (6.28%)	9.74 × 10^−4^
ABC transporters	ko02010	152 (1.15%)	397 (2.81%)	9.80 × 10^−4^
Peroxisome	ko04146	326 (2.47%)	929 (6.59%)	1.11 × 10^−3^
Glutathione metabolism	ko00480	248 (1.88%)	697 (4.94%)	2.38 × 10^−3^
Alanine, aspartate and glutamate metabolism	ko00250	210 (1.59)	594 (4.21%)	8.02 × 10^−3^

Pathways with *p*-value ≤ 0.05 are significantly enriched in DEGs.

**Table 4:** Enriched pathways containing down-regulated genes under Pb stress.

Pathway	Id	Number of DEGs with Pathway Annotations (13,204)	Number of Genes with Pathway Annotations (14,105)	*p*-Value
Ribosome	ko03010	3988 (30.20%)	8912 (63.18%)	8.16 × 10^−101^
Citrate cycle (TCA cycle)	ko00020	566 (4.29%)	1125 (7.98%)	1.48 × 10^−25^
Regulation of actin cytoskeleton	ko04810	584 (4.42%)	1262 (8.95%)	3.45 × 10^−16^
Gap junction	ko04540	269 (2.04%)	527 (3.74%)	1.67 × 10^−13^
Propanoate metabolism	ko00640	342 (2.59%)	722 (5.12%)	2.32 × 10^−11^
MAPK signaling pathway	ko04010	440 (3.33%)	1016 (7.20%)	7.54 × 10^−8^
Calcium signaling pathway	ko04020	284 (2.15%)	661 (4.69%)	5.06 × 10^−4^
d-Glutamine and d-glutamate metabolism	ko00471	33 (0.25%)	58 (0.41%)	8.42 × 10^−4^

Pathways with *p*-value ≤ 0.05 are significantly enriched in DEGs.

### 2.3. Validation by Real-Time RT-PCR

To validate the RNA-Seq technology, the expression levels of 12 candidate DEGs were detected using RT-qPCR (Table 5). The candidate genes selected for validation were associated with metal transporters, transcription factors and chelating compounds. Because AtNRT1.8 can mediate cadmium tolerance [22], we chose one NRT1 family gene, NRT1.2. The transcriptional expression patterns of nine genes (*comp152611_c0_seq1*, *comp100849_c0_seq1*, *comp123900_c0_seq1*, *comp160851_c0_seq1*, *comp142858_c0_seq3*, *comp130772_c0_seq3*, *comp1017906_c0_seq1*, *comp162326_c2_seq1* and *comp423843_c0_seq1*) based on RT-qPCR showed an approximate agreement with those of the RNA-Seq-based gene expression patterns (Table 5). However, three genes (*comp154333_c1_seq2*, *comp145580_c0_seq1* and *comp147199_c0_seq1*) did not show consistent expression levels between RT-qPCR and Illumina sequencing data (Table 5). The differences with respect to ratio and sensitivity between the two techniques may have led to the discrepancies [15].

**Table 5:** Validation of the RNA-Seq expression profiles of selected DEGs using qRT-PCR.

Transcript ID	Description	Fold by RNA-Seq	Fold by qPCR
*comp152611_c0_seq1*	Glutathione γ-glutamylcysteinyltransferase	2.64	1.89
*comp154333_c1_seq2*	Metallothionein-like protein type 2	1.90	3.68
*comp100849_c0_seq1*	WRKY transcription factor 22	0.44	0.62
*comp123900_c0_seq1*	EREBP-like factor	1.26	1.55
*comp145580_c0_seq1*	NAC domain-containing protein 90	3.58	5.22
*comp160851_c0_seq1*	Metal transporter Nramp2	1.29	1.43
*comp142858_c0_seq3*	Nitrate transporter 1.2	0.12	0.05
*comp147199_c0_seq1*	Glutathione *S*-transferase	6.73	8.33
*comp130772_c0_seq3*	Ethylene-responsive transcription factor ERF071	2.89	2.47
*comp1017906_c0_seq1*	Zinc finger CCCH domain-containing protein 40	9.86	8.73
*comp162326_c2_seq1*	Myb proto-oncogene protein. plant	1.27	1.38
*comp423843_c0_seq1*	Transcription factor bHLH104	2.41	3.04

### 2.4. Analysis of Differential Gene Expression

In a previous study, no morphological differences were observed between the Pb-stressed seedling and control [23]. To elucidate the expression levels of functional genes and transcription factors under Pb stress, 3869 significantly changed genes were identified between control and Pb-stressed libraries using the reads per kilobase transcriptome per million mapped reads method. Among these, the number of up- and down-regulated genes was 1850 and 2019, respectively.

Some significant differential gene expressions were analyzed (Table S3). Metallothioneins (MTs) and phytochelatins (PCs) are two types of protein molecules in the biosynthesis of chelating compounds [24]. Class II MTs typically contain four categories (types 1–4) [25], and PCs are non-protein cysteine-rich oligopeptides that are capable of binding to various metals [26]. In the present study, two phytochelatin synthase DEGs, two metallothionein-like protein type 2 DEGs and one metallothionein-like protein type 3 DEG were found in the DEG analysis (Table S3). In *A. corniculatum*, the expression levels of *AcMT* genes were also induced by a certain concentration range of Pb stress compared with the control [27]. Moreover, MT genes were also affected by heavy metals in *Iris lacteal* [19], *Brassica juncea* [28] and *Brassica campestris* [29]. PCs have been demonstrated to be important in heavy metal detoxification and may be used as candidate genes in the phytoremediation of heavy metals [24]; they were found in alfalfa [20], *Dianthus carthusianorum* [30] and *Lotus japonicus* [31,32].

Transmembrane metal transporters are assumed to play key roles in heavy metal transport and detoxification [33]. ABC family transporters are the important heavy metal transporters [34]. In our study, the expression levels of ABC transporters were inhibited or induced. Another PDR-type ABC transporter, *OsPDR9*, is induced by heavy metals in rice roots [35]. *AtPDR8*, which encodes an ABC transporter, is a cadmium extrusion pump involved in heavy metal resistance [36]. Two zinc transporters (*ZIP1* and *ZIP3*) showed inducible higher expression levels. However, *ZIP8* and *ZIP9* were down-regulated under Pb stress (Table S3). In rice, five *ZIP* transporter genes have been reported [37]. *OsZIP1* is strongly inhibited by cadmium, whereas *OsZIP3* is inhibited to a lesser extent by calcium [38]. *OsZIP4* may be involved in the translocation of zinc [39]. It would be interesting to know the function of the *ZIP* gene family in *Louisiana iris*. Another ferrous iron transporter, iron-regulated protein 3 (IRT3), was down-regulated under Pb stress (Table S3). But *IRT1* is more highly expressed in *Solanum nigrum* than in *Solanum torvum* [20]. In *A. thaliana*, the IRT1 protein mediates the uptake of heavy metals [40]. In *Louisiana iris* root, Pb may be a substrate of the *IRT3* protein.

Three metal transporters, a copper transporter, *CTR2*, a magnesium transporter, *MGT* and a heavy metal P-type ATPase (*HMA5*), showed constitutive expression levels. However, their transcript levels significantly increased under Pb-stress conditions (Table S3). In *A. thaliana*, a five-member family of copper transporters (*COPT1-5*) was identified, and *COPT1* and *COPT2* were down-regulated under copper excess [41]. COPT5 acts as a copper exporter and is involved in the inter-organ reallocation of copper [42]. AtMRS2-11, a putative magnesium transporter, plays an important role in transporting magnesium in the chloroplast membrane system [43]. Moreover, a root-expressed magnesium transporter, *MRS2-7*, allows *A. thaliana* to grow in low-magnesium environments [44]. For HMA proteins, AtHMA2 is involved in cytoplasmic zinc homeostasis [45]. TcHMA3 is responsible for cadmium tonoplast-localized transportion [46]. TcHMA4 could display cadmium tolerance in the *Thlaspi caerulescens* cadmium hyperaccumulator [47]. AtHMA5 interacts with ATX1-like copper chaperones and functions in copper compartmentalization and detoxification [48]. High expression levels of *CTR2*, *MGT* and *HMA5* in *Louisiana iris* roots may mediate Pb uptake and transport in plants.

Transcription factors are very important in gene expression regulation [49]. In our results, the transcript expression levels of one *bHLH*, five *ERFs* and one *DREB* were affected by Pb stress, meaning that transcription factors and plant heavy metal stress tolerance have a close relationship. These results had been found in previous studies. AtbHLH29 is involved in controlling iron acquisition [50]. Transgenic plants overexpressing *FIT/AtbHLH38* or *FIT/AtbHLH39* could exhibit more cadmium tolerance [51]. For the *MYBs* and *ERFs*, there were six DEGs, including up- and down-regulated genes, in radish under Pb stress [15]. *DREB* genes may play a role in copper tolerance in rice [52], and *LbDREB* was found to enhance copper tolerance in transgenic tobacco [53].

Pb stress can affect the activity of peroxidase, superoxide dismutase, and catalase in plants [54]. One peroxidase, two superoxide dismutases, and one catalase were highly expressed in *Louisiana iris* roots. The expression patterns were consistent with the physiological results (data not shown).

## 3. Experimental Section

### 3.1. Sampling

In our previous study, *Louisiana iris* hybrid “Professor Neil” was confirmed to be a tolerant cultivar that can be used for the phytoremediation of contaminated environments [23]. When the plants were under a 200 mg/L Pb(NO_3_)_2_ stress treatment, we did not observe any obvious damage. However, visible stress phenotypes (data not shown) can been seen at 400 mg/L Pb(NO_3_)_2_. Therefore, 200 mg/L Pb(NO_3_)_2_ stress was selected as the treatment. The root is not only the first tissue damaged but also the main tissue bearing the Pb stress. Roots were, therefore, chosen as the most suitable tissue for this experiment. The seedlings were prepared as described by Gu *et al.* [19]. The 200 mg/L Pb(NO_3_)_2_ stress treatment was given to tissue culture seedlings after they reached 10 cm height in the greenhouse of the Institute of Botany, Jiangsu Province and the Chinese Academy of Sciences (Nanjing, China). Fresh roots of seedlings (three plants per sample) were harvested at two times (0 and 24 h). Samples were immediately frozen in liquid nitrogen until used.

### 3.2. cDNA Library Preparation and Sequencing

Total RNA was isolated with Trizol reagent (Life Technologies, Inc., Grand Island, NY, USA) following the manufacturer’s instructions [5]. Potentially contaminating DNA was eliminated from the total RNA using DNaseI (RNase-free New England Biolabs, Ipswich, MA, USA). RNA quality was confirmed using an Agilent Technologies 2100 Bioanalyzer (Agilent Technologies, Palo Alto, CA, USA), and the RIN values were greater than 7. All of the samples had density absorption ratios A260/A280 between 1.9 and 2.1, and were adjusted to the same RNA concentration. First-strand cDNA was synthesized using the SMART cDNA Synthesis Kit (Clontech Laboratories, Inc., Mountain View, CA, USA). After the second-strand cDNA synthesis reaction, the cDNAs were purified using a QiaQuick PCR extraction kit (Qiagen, Hilden, Germany) and connected with sequencing adapters. The PCR amplifications were selected as templates. The resulting library was sequenced using the Illumina HiSeqTM 2000 platform (Illumina, San Diego, CA, USA).

### 3.3. Transcriptome Sequencing Results Analysis

Raw sequence processing and *de novo* assembly were performed following the procedures described by Wang *et al.* [15]. After the adapters and low quality tags were removed, high-quality clean reads were obtained and assembled using Trinity (trinityrnaseq_r2012-10-05) [55]. Subsequently, unigenes were acquired using the TIGR Gene Indices clustering tools (http://www.tigr.org/tdb/tgi.shtml) [56]. Furthermore, the unigenes were annotated using the BLASTx algorithm against Swiss-Prot, TrEMBL, NCBI, CDD, Pfam and KOG databases with an *E*-value ≤10^−5^. The Blast2Go program was used to analyze GO annotations of unigenes. KOG and KEGG were also used to complement GO functional characterizations and determine the sequence directions of the unigenes.

### 3.4. Real-Time RT-PCR

Total RNA was isolated from young *Louisiana iris* roots using the RNAiso reagent (TaKaRa, Bio, Tokyo, Japan). RT-qPCR assays were performed on a Mastercycler^®^ep *realplex* Real-Time System (Eppendorf, Hamburg, Germany) platform using the SYBR^®^ Premix *Ex Taq*™ II (Perfect Real Time) (TaKaRa, Bio, Tokyo, Japan). UBC (Table S4) was used as the reference gene described by Gu *et al.* [57]. The amplifications were performed as follows: 60 s at 95 °C for denaturation, 40 cycles of 15 s at 95 °C, 30 s at 55 °C, and 30 s at 72 °C [58]. Each RT-qPCR reaction was performed in three biological replicates. The 2^−∆∆*C*t^ method was applied for mean values [59].

### 3.5. Analysis of Gene Differential Expression

To show the differential gene expression levels between control and Pb-stressed roots, we used the reads per kilobase transcriptome per million mapped reads method to calculate the gene expression levels [60]. Significantly, DEGs were identified by estimating the false discovery rate (*p* ≤ 0.001) and absolute values of the log2Ratio (Pb/control) ≥ 1.

## 4. Conclusions

In summary, the root transcriptome of *Louisiana iris* was first characterized by Illumina sequencing. Based on a digital gene expression analysis, many genes that are involved in Pb-stress responses were found. The rich genetic and genomic resources in the transcriptomic data lay a foundation for studying the molecular mechanisms of *Louisiana iris* tolerance to Pb stress.

## Supplementary Materials

Supplementary materials can be found at http://www.mdpi.com/1422-0067/16/12/26084/s1.
