# Supplementary material for: Transcriptome Profiling of Louisiana iris Root and Identification of Genes Involved in Lead-Stress Response
Source: Int J Mol Sci. 2015 Nov 25;16(12):28087–97. doi: 10.3390/ijms161226084 (PMC4691031; doi:10.3390/ijms161226084)
Supplement: Supplementary file 1 [file ijms-16-26084-s001.zip › ijms-89590-Supplementary Information.pdf]

Supplementary Materials: Transcriptome Profiling of *Louisiana iris* Root and Identification of Genes Involved in Lead-Stress Responses Using next Generation Sequencing

Songqing Tian, Chunsun Gu, Liangqin Liu, Xudong Zhu, Yanhai Zhao and Suzhen Huang

Table S1. The enriched GO terms of up-regulated DEGs. Please see Table S1 (Excel).

Table S2. The enriched GO terms of down-regulated DEGs. Please see Table S2 (Excel).

Table S3. Enriched pathways of genes down-regulated by Pb stress.

Transcript ID	Foldchange	Description
	**Biosynthesis of chelating compounds**	
comp152611_c0_seq1	1.4021	glutathione gamma-glutamylcysteinyltransferase
comp473184_c0_seq	1.2303	glutathione gamma-glutamylcysteinyltransferase 2
comp142955_c0_seq5	0.2476	Metallothionein-like protein type 2
comp183887_c0_seq1	−1.7325	Metallothionein-like protein type 2
comp57369_c0_seq1	0.3975	Metallothionein-like protein type 3
	**Activation of metal transporters**	
comp103175_c0_seq1	2.8875	ABC transporter B family member 1
comp1035409_c0_seq1	2.4854	ABC transporter B family member 19
comp104944_c0_seq1	−0.6374	ABC transporter G family member 24
comp149639_c0_seq1	3.0764	ABC transporter A family member 1
comp105611_c0_seq1	1.3726	ABC transporter A family member 9
comp106081_c0_seq1	0.5025	ABC transporter G family member 28
comp725811_c0_seq1	4.3923	ABC transporter C family member 5
comp787957_c0_seq1	3.8413	ABC transporter G family member 12
comp264126_c0_seq1	3.7134	ABC transporter B family member 3
comp1586198_c0_seq1	3.5546	ABC transporter C family member 7
comp26091_c0_seq1	−3.3074	ABC transporter F family member 2
comp267439_c0_seq1	2.3451	Zinc transporter 1
comp127056_c0_seq1	1.9794	Zinc transporter 2
comp389851_c0_seq1	−3.1964	Zinc transporter 8
comp1341294_c0_seq1	−2.1155	Zinc transporter 9
comp295312_c0_seq1	−2.0000	Iron-regulated protein 3
comp7283_c0_seq1	1.5546	Copper transporter 2
comp1480545_c0_seq1	2.1155	Magnesium transporter mgtE
comp465044_c0_seq1	1.4203	Copper-transporting ATPase HMA5
	**Modulation of transcription factors**	
comp423843_c0_seq1	1.2675	Transcription factor bHLH104
comp129460_c0_seq1	−1.1117	Ethylene-responsive transcription factor 2
comp130772_c0_seq3	1.5299	Ethylene-responsive transcription factor ERF071
comp52367_c0_seq1	3.5206	Ethylene-responsive transcription factor ERF094
comp93080_c0_seq2	2.9668	Ethylene-responsive transcription factor 14
comp95061_c0_seq1	1.0359	Ethylene-responsive transcription factor 5
comp1824_c0_seq2	3.3626	Dehydration-responsive element-binding protein 2A
	**Antioxidant-related genes**	
comp105748_c0_seq1	4.3399	Secretoryperoxidase
comp135589_c0_seq1	2.7327	Ascorbateperoxidase
comp1264311_c0_seq1	−2.7370	L-ascorbate peroxidase 5
comp143076_c0_seq1	2.2541	Peroxidase
comp1017077_c0_seq1	1.7244	Superoxide dismutase [Cu-Zn]
comp1332564_c0_seq1	1.2095	Superoxide dismutase [Fe]
comp128035_c0_seq2	2.4034	Catalase

Table. S4. The primers for candidate genes that respond to heavy metal Pb.

Primer	Sequence (5′–3′)
comp152611_c0_seq1	CAAGTATCCTCCTCACTGGGT<br>CACTCCACCACAAGATGAAAG
comp154333_c1_seq2	CGAAGGATTTGAGACTGGTG<br>TCATGGCCTCGTTTATTTTA
comp100849_c0_seq1	GGAAGTACGGACAGAAGCCC<br>GCGGTGTAGGTAATGATGAA
comp123900_c0_seq1	GGGACTACGCCAGGCTAAACT<br>TAAGGTGGAAGCTCTCCGACT
comp145580_c0_seq1	GCTCCCTGTGGGCTATCGCTT<br>AGCTCCCCAATGACTTTGCGG
comp160851_c0_seq1	GGTCCAAAACCATAAAGCAGG<br>AAGGACAGGAAAAAGCATAGC
comp142858_c0_seq3	TCGGCACCGTCATCTTCATTA<br>TAGCTCCTTCGGGTTCTCAGG
comp147199_c0_seq1	TTGCTCTCGGTTGCTATCTTG<br>TCTGGCAGTGCATCTTTCACT
comp130772_c0_seq3	ATGTGTGGAGGTGCTATTATT<br>CTTCGGCTTAGGCTTCAGTTT
comp1017906_c0_seq1	ACCACCTGCAGATCAATCCAT<br>GCACCTTCTCTCTTTGCGAAA
comp162326_c2_seq1	AACTACCTCCGCCCCGACAT<br>ATTGAGGGTTGCGGACTTGA
comp423843_c0_seq1	AGATGATTCCTTTGGAGATGG<br>TTGACTCAGAACACGGATAGC
UBC	TCTCGCTTGTCCGGTTTGTG<br>ACCTTGGGTGGCTTGAATGG
